# Panic, Psycho-Behavioral Responses, and Risk Perception in the Earliest Stage of the COVID-19 Pandemic in China

**DOI:** 10.3389/fmed.2022.766842

**Published:** 2022-02-25

**Authors:** Weiyu Zhang, Changqing Zou, Kristin K. Sznajder, Can Cui, Jiahui Fu, Shan He, Qinqi Peng, Qiongli Chen, Xiaoshi Yang

**Affiliations:** ^1^Department of Social Medicine, College of Health Management, China Medical University, Shenyang, China; ^2^Department of Humanities and Social Sciences, China Medical University, Shenyang, China; ^3^Department of Public Health, Pennsylvania State University College of Medicine, Hershey, PA, United States

**Keywords:** panic, behavior, COVID-19, online survey, mental health

## Abstract

**Background:**

Coronavirus Disease-19 (COVID-19), a rising global pandemic, has triggered psychological crises among the public. Panic, a severe symptom of mental disorders, is increasing in the public in China and it is urgent to provide research for intervention development.

**Objectives:**

This study aimed to assess the prevalence of public panic in China during the earliest stage of the COVID-19 pandemic and to explore the associated psychological behavioral responses and public's risk perception of the pandemic.

**Methods:**

A cross-sectional study using a web-based survey with convenience sampling was conducted with 2,484 participants nationally from February 11 to February 24, 2020 in China. A self-developed questionnaire was applied to assess the prevalence of public panic and its associated factors. Multivariable logistic regression analysis was applied to assess the risk and protective factors of public panic.

**Results:**

There were 23.39% (581/2,484) of the participants who reported experiencing panic during the earliest stage of the COVID-19 pandemic. Taking temperature repeatedly, being nervous in a crowd, being suspicious of infection in the family, being worried about the future, and worries about high infectivity of the COVID-19, lack of effective therapies, and wide impact of the COVID-19 pandemic increased the odds of public panic. Whereas, avoiding gatherings during holidays was negatively associated with the odds of public panic.

**Conclusions:**

Psycho-behavioral responses were closely associated with public panic during the earliest stage of the COVID-19 pandemic in China. Defusing excessive health-related worries, the guidance of appropriate self-protective behaviors, strengthening of health education in communities, and available treatment for mental disorders should be adopted to monitor the psychological responses and to guide the behaviors of the public.

## Introduction

Coronavirus Disease-19 (COVID-19) has become an unprecedented enormous challenge facing mankind. It has not only affected human health and global development but has also caused social shutdowns and major economic damage. COVID-19 has not only caused a public health crisis, but it has also resulted in an information crisis (1–4). To slow the spread of COVID-19 in China, compulsory actions comprising the establishment of targeted hospitals, restrictions on traffic, community isolation, etc., have been taken (5). However, growing research has found that negative psychological responses, such as indifference, paranoia, sadness, fear, and anxiety, have increased during the COVID-19 pandemic (6–8). Mental health crises are also closely associated with the experience of public health emergencies (9–11). The decline in the public's mental wellbeing especially the high prevalence of psychological distress and panic has raised considerable concerns among medical fields, which will require urgent assessment and management (12–15).

The mental wellbeing of the public during the COVID-19 pandemic has highlighted multidimensional risk factors. Research on the behavioral immune system indicates that pandemics can induce extreme responses, such as persistent doubting and checking and excessive sensations about minor bodily ailments (16, 17). As the cognitive account of panic attacks presented, panic could result from a misinterpretation of external events, and interventions providing accurate and reliable information may be effective to prevent public panic (18–20). Additionally, the danger-laden schemas in mental disorders also suggest the appraisal of events as threatening because of the high-risk perception (21–23). In summary, panic can be triggered by physiological, psychological, and environmental factors during the unexpected suffering experiences.

Most people have paid attention to public health information about the pandemic and personal protection, adopting social distancing measures, regular hand washing and mask wearing, and canceling their social and travel plans. While public health measures are essential to fight against COVID-19, these measures have changed the public's sense of security, which have led to higher levels of stress, emotional disturbances, irrational anger, panic, impulsivity, anxiety, and depression among the public during the initial phase of the COVID-19 pandemic in China. Further, home confinement, being worried about family members exposed to COVID-19, the shortage of personal protective equipment, uncertainties of education or work progression, and the inaccuracy of the health information have exacerbated negative mental health (24–29). A stimulus-organism-response model proposed by a recent study indicates that information-avoidance behavior and perceived information overload are associated with sadness, anxiety, and cognitive dissonance during the COVID-19 pandemic (30–33). A variety of adverse conditions, such as public panic, are critical to be acknowledged and measured.

Additionally, behavioral changes are common during public health emergencies. For example, the social restrictions were consistently related to changes in behaviors, such as reduced physical activity, using hand sanitizer, panic buying and hoarding, and poor eating behaviors, during the COVID-19 pandemic (34–38). The Health Belief Model indicates that cognitive processing has a strong connection with the perception of risks and protective behaviors (39, 40). Furthermore, the integrative cognitive model of panic attacks and the neuroanatomical theory suggest that the cognition and apprehension of trigger stimulus are associated with panic (18, 23, 41, 42). Additionally, anxiety and cognitive risks are closely associated with protective behaviors (e.g., avoiding crowds, disinfecting the living environment) during the epidemic, especially during the early stage (43). Psycho-behavioral responses and panic share a close and significant relationship during the unexpected pandemic and accompany changes in daily life.

Various psychological responses could be correlated with public panic during the COVID-19 pandemic for personal protection, such as being nervous in a crowd, being worried about the future, being afraid of the recurrence of the COVID-19 pandemic, and being suspicious that infected people exist are around. Plenty of excessive protective behaviors may also significantly impact public panic, such as repeated temperature measurement and avoidance of gatherings during the holidays. Therefore, exploring the associations between the psycho-behavioral responses, the risk perception of the pandemic, and panic is crucial for the enhancement of public mental health during the COVID-19 pandemic.

## Materials and Methods

### Participants, Procedure, and Ethics Statement

From February 11 to 24, 2020, a cross-sectional survey was conducted in 2,484 participants using convenience sampling in mainland China. A link of the self-administrated questionnaire developed by the Environmental Health Institute at China Medical University was distributed via WeChat, one of the most popular social media platforms in China. The inclusion criteria of participants were as follows: at least 18 years of age; able to comprehend and complete the online questionnaire in Chinese independently; willing to participate in the study and; and able to provide signed informed consent. The exclusion criteria of the participants were as follows: having a history of the severe psychological disease (e.g., schizophrenia, bipolar disorder, mental disorders caused by epilepsy); having current psychological therapy; and having been diagnosed with diseases which limited the participation in this survey.

The validated questionnaire contained self-developed questions about panic, psycho-behavioral responses to the COVID-19 pandemic, and the risk perception of the COVID-19 pandemic. The questionnaire took about 20 min to complete. All of the procedures of this survey followed the Helsinki Declaration as revised in 1989 and the protocol authorized by the Ethics Committee of China Medical University (ID: 2020048).

### Demographic Characteristics of the Participants

The gender (male, female), age, marital status (married, other), occupation (government worker/healthcare worker/employee of an enterprise/student/other [e.g., commercial personnel, soldier, teacher, etc.]), education (college and below, bachelor's degree, master's degree, or above), and monthly income (≤ 5,000 yuan [ ≤ US $725.19], 5,001–10,000 yuan [US $725.34–1,450.39], and >10,000 yuan [>US $1,450.39]) were collected as demographic characteristics.

### Assessment of Public Panic

A self-developed question was applied to measure the public panic among the public. Public panic was collected using a yes or no question: “I was panicked during the COVID-19 pandemic” with choices including “yes” or “no”.

### Assessment of Psycho-Behavioral Responses

Behaviors in the past 2 weeks, such as “Taking temperature repeatedly”, “being nervous in a crowd”, “being suspicious of infection in the family”, “being worried about the future”, “being afraid of the recurrence of the COVID-19 pandemic”, “being constantly reminded of the COVID-19 pandemic by the surroundings”, “being suspicious that infected people are around”, and “avoiding gatherings during the holidays” comprised the collected psycho-behavioral responses. The psycho-behavioral responses were assessed by self-developed “yes” or “no” questions.

### Assessment of Risk Perception of the COVID-19 Pandemic

The perception of risk during the COVID-19 pandemic was assessed with a multiple-choice question. The choices included worries about high infectivity of COVID-19; lack of effective therapies; the wide impact of the COVID-19 pandemic; lack of medical supplies and healthcare workers; sequelae after recovery from COVID-19; extensive announcements, rumors, and nervousness; extensive media coverage; and news of the pandemic on the internet.

### Statistical Analyses

This study carried out the statistical analyses with SPSS version 23.0 statistical software for Windows (IBM Corporation). The relationship between public panic and other variables was explored with chi-square tests. The risk factors and preventive factors of public panic were explored with multivariable logistic regression analysis. Associations were considered significant when there was a two-tailed *p* < 0.05.

## Results

### Demographic Characteristics and Public Panic Distribution of the Public

The prevalence of public panic during the earliest stage of the COVID-19 pandemic was 581/2,484 (23.39%). The results from the chi-square tests on public panic are shown in Table 1. The participants who were not married (1,240/2,484, 49.92%) had a higher prevalence of panic (*p* = 0.022). Differences in participants panic by education level (*p* = 0.034) and occupations (*p* < 0.001) were also significant.

**Table 1:** The distribution of public panic (*N* = 2,484).

**Variables**	**Total** **[n, (%)]**	**Panic** **[n, (%)]**	**No Panic** **[n, (%)]**	**X^**2**^**	***P***
**Demographic characteristics**					
**Gender**				**1.048**	**0.165**
Male	934 (37.60)	208 (35.80)	726 (38.15)		
Female	1,550 (62.40)	373 (64.20)	1,177 (61.85)		
**Age (years)**				**0.029**	**0.451**
≤ 35	1,474 (59.34)	343 (59.04)	1,131 (59.43)		
>35	1,010 (40.66)	238 (40.96)	772 (40.57)		
**Marital status**				**8.071**	**0.003**
Married	1,244 (50.08)	261 (44.92)	983 (51.66)		
Other	1,240 (49.92)	320 (55.08)	920 (48.34)		
**Occupation**				**21.396**	**<0.001**
Government worker/ employee of enterprises	507 (20.41)	132 (22.72)	375 (19.71)		
Health care worker	336 (13.53)	80 (13.77)	256 (13.45)		
Student	954 (38.41)	178 (30.64)	776 (40.78)		
Other (e.g., commercial personnel, soldier, teacher, etc.)	687 (27.66)	191 (32.87)	496 (26.06)		
**Education**				**6.775**	**0.034**
College and below	289 (11.63)	85 (14.63)	204 (10.72)		
Bachelor's degree	1,395 (56.16)	319 (54.91)	1,076 (56.54)		
Master's degree and above	800 (32.21)	177 (30.46)	623 (32.74)		
**Monthly income (¥^a^)**				**1.097**	**0.578**
≤ 5,000	995 (40.06)	222 (38.21)	773 (40.62)		
5,001–10,000	884 (35.59)	212 (36.49)	672 (35.31)		
>10,000	605 (24.36)	147 (25.30)	458 (24.07)		
**Psycho-behavioral responses**					
**Taking temperature repeatedly**				**41.375**	**<0.001**
Yes	990 (39.86)	298 (51.29)	692 (36.36)		
No	1,494 (60.14)	283 (48.71)	1,211 (63.64)		
**Being nervous in a crowd**				**70.421**	**<0.001**
Yes	1,600 (64.41)	459 (79.00)	1,141 (59.96)		
No	884 (35.59)	122 (21.00)	762 (40.04)		
**Being suspicious of infection in the family**				**88.664**	**<0.001**
Yes	869 (34.98)	298 (51.29)	571 (30.01)		
No	1,615 (65.02)	283 (48.71)	1,32 (69.99)		
**Being worried about the future**				**123.733**	**<0.001**
Yes	1,196 (48.15)	397 (68.33)	799 (41.99)		
No	1,288 (51.85)	184 (31.67)	1,104 (58.01)		
**Being afraid of the recurrence of the COVID-19 pandemic**				**22.305**	**<0.001**
Yes	611 (24.6)	100 (17.21)	511 (26.85)		
No	1,873 (75.4)	481 (82.79)	1,392 (73.15)		
**Being constantly reminded of the COVID-19 pandemic by the surroundings**				**34.579**	**<0.001**
Yes	589 (23.71)	85 (14.63)	504 (26.48)		
No	1,895 (76.29)	496 (85.37)	1,399 (73.52)		
**Being suspicious that infected people are around**				**47.189**	**<0.001**
Yes	712 (28.66)	101 (17.38)	611 (32.11)		
No	1,772 (71.34)	480 (82.62)	1,292 (67.89)		
**Avoiding gatherings during the holidays**				**11.705**	**0.001**
Yes	2,328 (93.72)	527 (90.71)	1,801 (94.64)		
No	156 (6.28)	54 (9.29)	102 (5.36)		
**Risk perception of the COVID-19 pandemic**					
**Worries about high infectivity of the COVID-19**				**22.863**	**<0.001**
Yes	2,036 (81.96)	515 (88.64)	1,521 (79.93)		
No	448 (18.04)	66 (11.36)	382 (20.07)		
**Lack of effective therapies**				**25.131**	**<0.001**
Yes	1,780 (71.66)	464 (79.86)	1,316 (69.15)		
No	704 (28.34)	117 (20.14)	587 (30.85)		
**Wide impact of the COVID-19 pandemic**				**20.311**	**<0.001**
Yes	1,710 (68.84)	444 (76.42)	1,266 (66.53)		
No	774 (31.16)	137 (23.58)	637 (33.47)		
**Lack of medical supplies and health care workers**				**2.088**	**0.157**
Yes	1,540 (62.00)	375 (64.54)	1,165 (61.22)		
No	944 (38.00)	206 (35.46)	738 (38.78)		
**Sequelae after recovery from COVID-19**				**20.037**	**<0.001**
Yes	719 (28.95)	211 (36.32)	508 (26.69)		
No	1,765 (71.05)	370 (63.68)	1,395 (73.31)		
**Extensive announcements, rumors, and nervousness**				**25.596**	**<0.001**
Yes	350 (14.09)	119 (20.48)	231 (12.14)		
No	2,134 (85.91)	462 (79.52)	1,672 (87.86)		
**Extensive media coverage about the pandemic**				**22.492**	**<0.001**
Yes	276 (11.11)	96 (16.52)	180 (9.46)		
No	2,208 (88.89)	485 (83.48)	1,723 (90.54)		
**News of the pandemic on the internet**				**13.992**	**<0.001**
Yes	436 (17.55)	132 (22.72)	304 (15.97)		
No	2,048 (82.45)	449 (77.28)	1,599 (84.03)		

a *1 ¥ = US $0.15. X^2^: A measure of the difference between the observed and expected frequencies of the outcomes of a set of events or variables. P: The possibility the data could have occurred under the null hypothesis*.

### Psycho-Behavioral Responses

**Figure 1:**
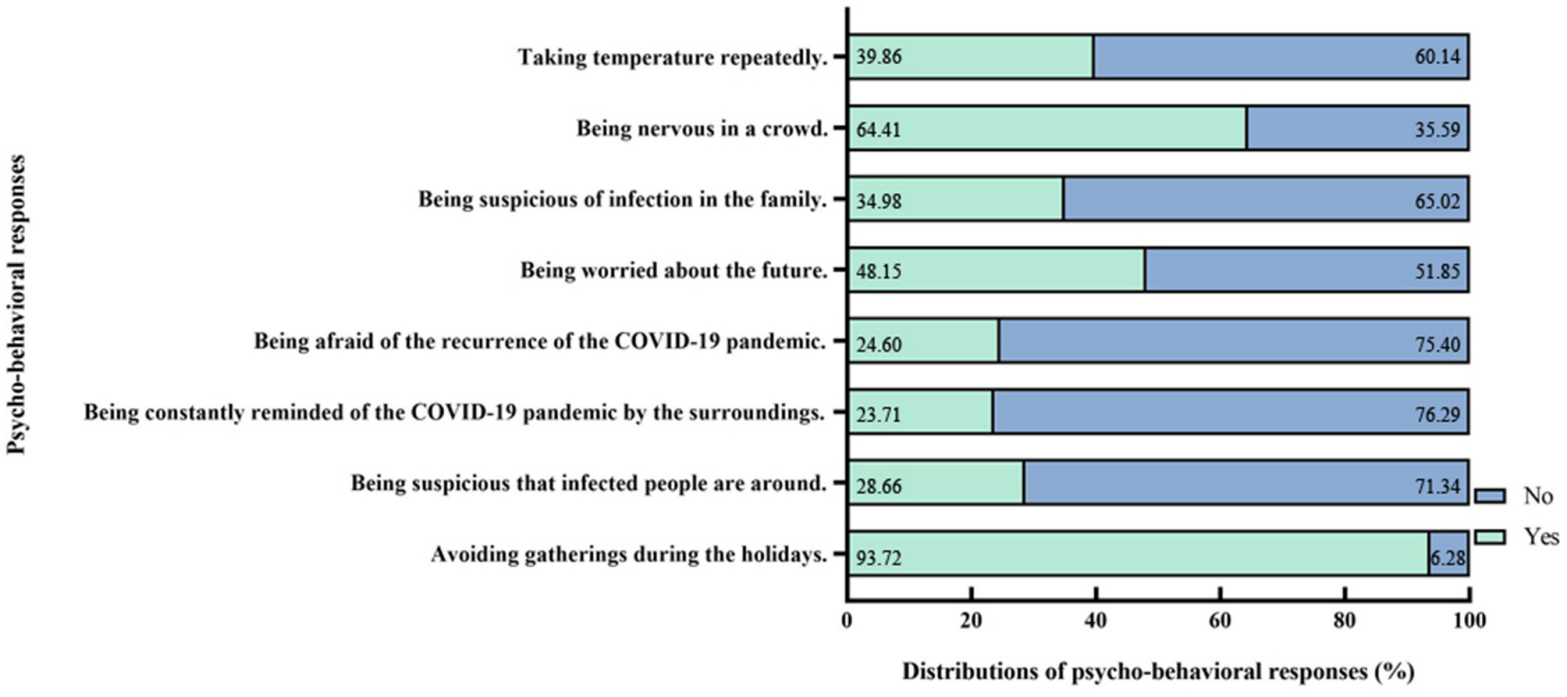
Psycho-behavioral responses and distributions of panic.

**Figure 2:**
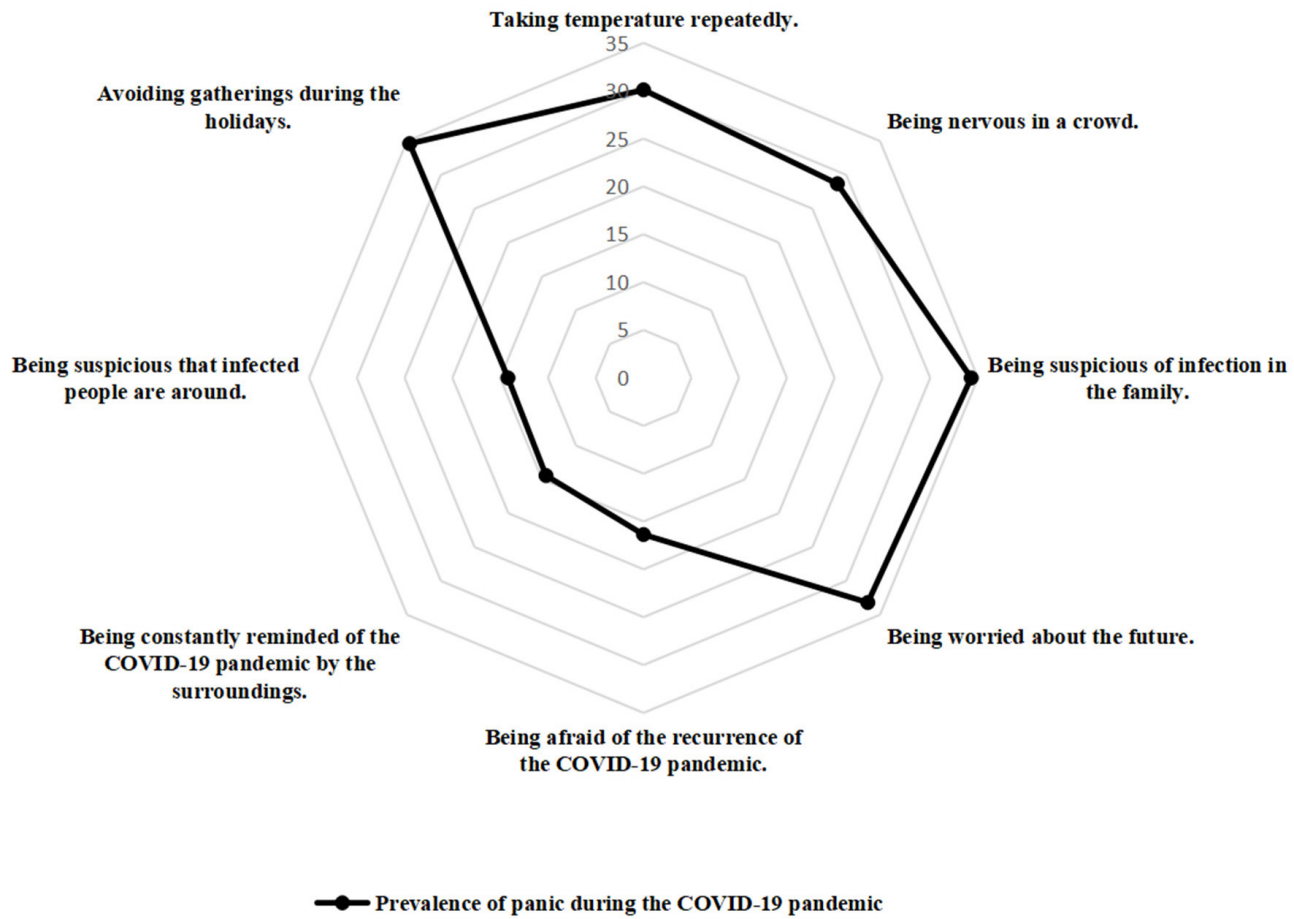
The radar map of the distribution of the public panic (psycho-behavioral responses).

The psycho-behavioral responses and corresponding prevalence of public panic are also presented in Table 1; Figures 1, 2. Taking temperature repeatedly (990, 39.86%), being nervous in a crowd (1,600, 64.41%), being suspicious of infection in the family (869, 34.98%; *p* < 0.001), being worried about the future (1,196, 48.15%), being afraid of the recurrence of the COVID-19 pandemic (611, 24.60%), being constantly reminded of the COVID-19 pandemic by the surroundings (589, 23.71%), and being suspicious that infected people are around (712, 28.66%; *p* < 0.001), and avoiding gathering during the holidays (2,328, 93.72%; *p* = 0.001) were all significant factors of public panic.

### Risk Perception of the COVID-19 Pandemic

**Figure 3:**
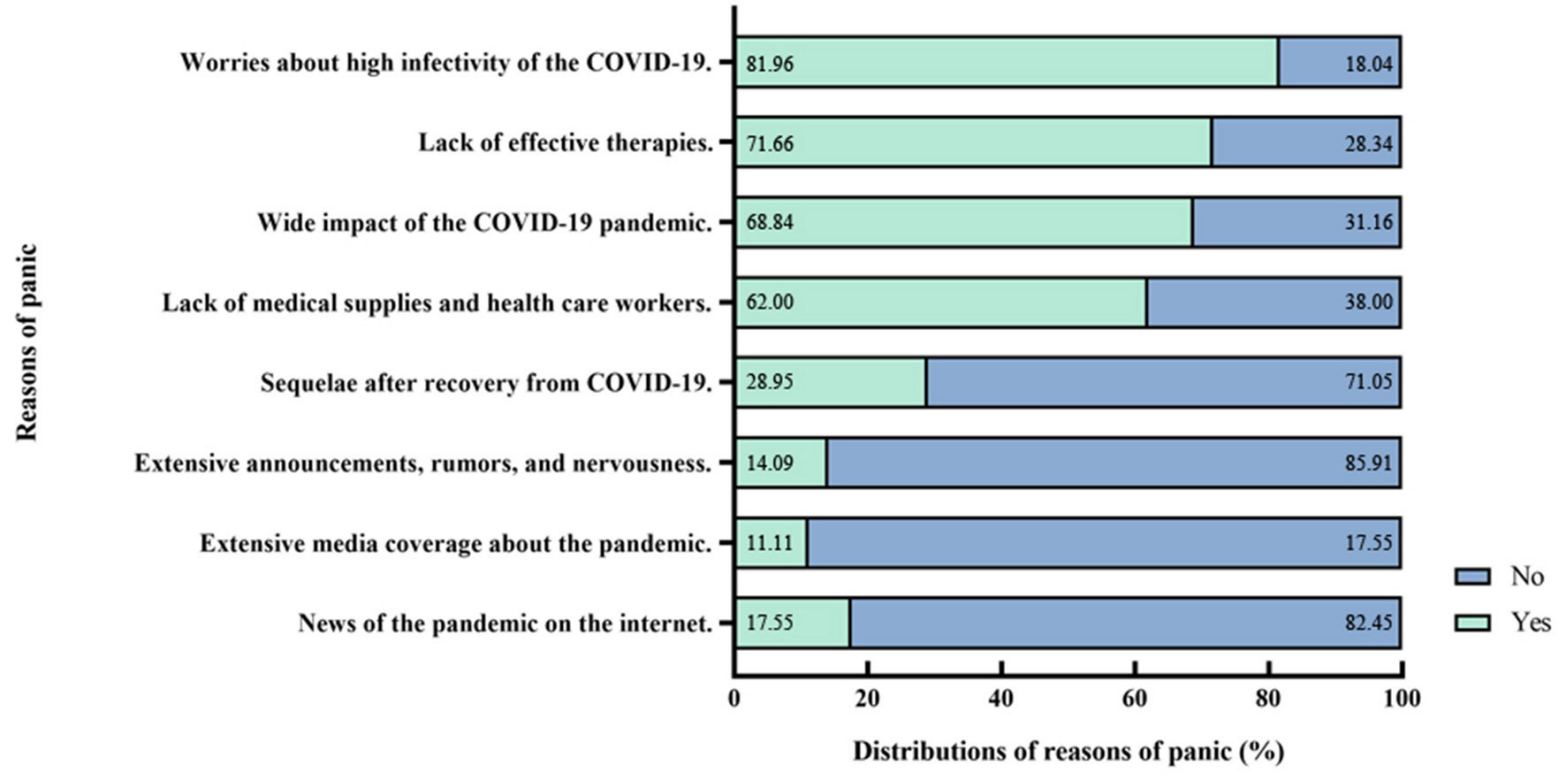
Risk perception of the Coronavirus Disease-2019 (COVID-19) pandemic and distributions of panic.

**Figure 4:**
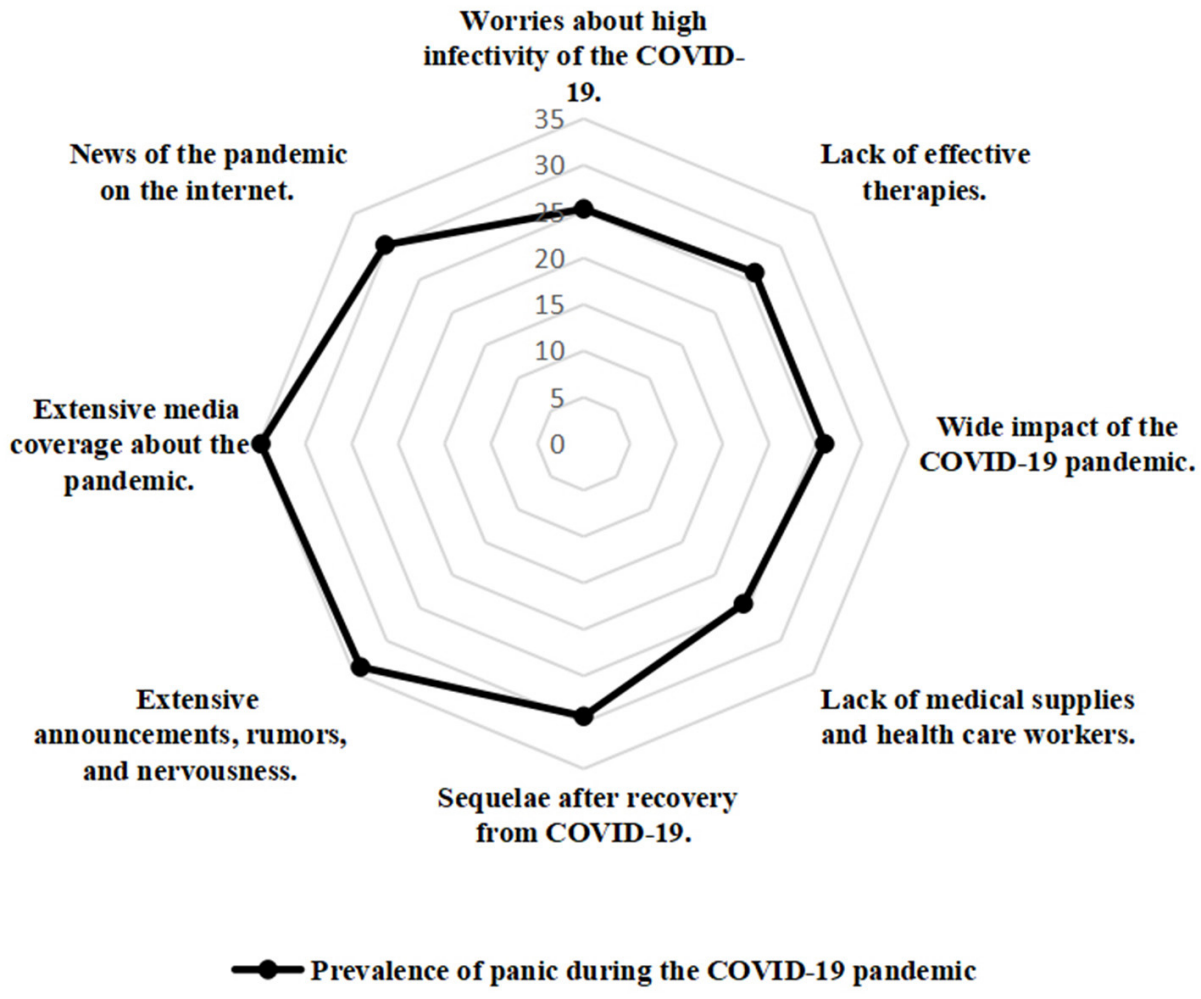
The radar map of the distribution of the public panic (risk perception of the Coronavirus Disease-2019 (COVID-19) pandemic).

Participates' risk perception of the COVID-19 pandemic and the corresponding prevalence of panic are shown in Table 1; Figures 3, 4. Worries of high infectivity of COVID-19 (2,036/2,484, 81.96%); lack of effective therapies (1,780, 71.66%); wide impact of the COVID-19 pandemic (1,710/2,484, 68.84%); sequelae after recovery from COVID-19 (719/2,484, 28.95%); extensive announcements, rumors, and nervousness (350, 14.09%; *p* < 0.001); extensive media coverage about the pandemic (276/2,484, 11.11%); and news of the pandemic on the internet (436/2,484, 17.55%; *p* < 0.001) were significantly associated with public panic.

### Risk Factors and Associated Factors of Public Panic During the COVID-19 Pandemic

**Figure 5:**
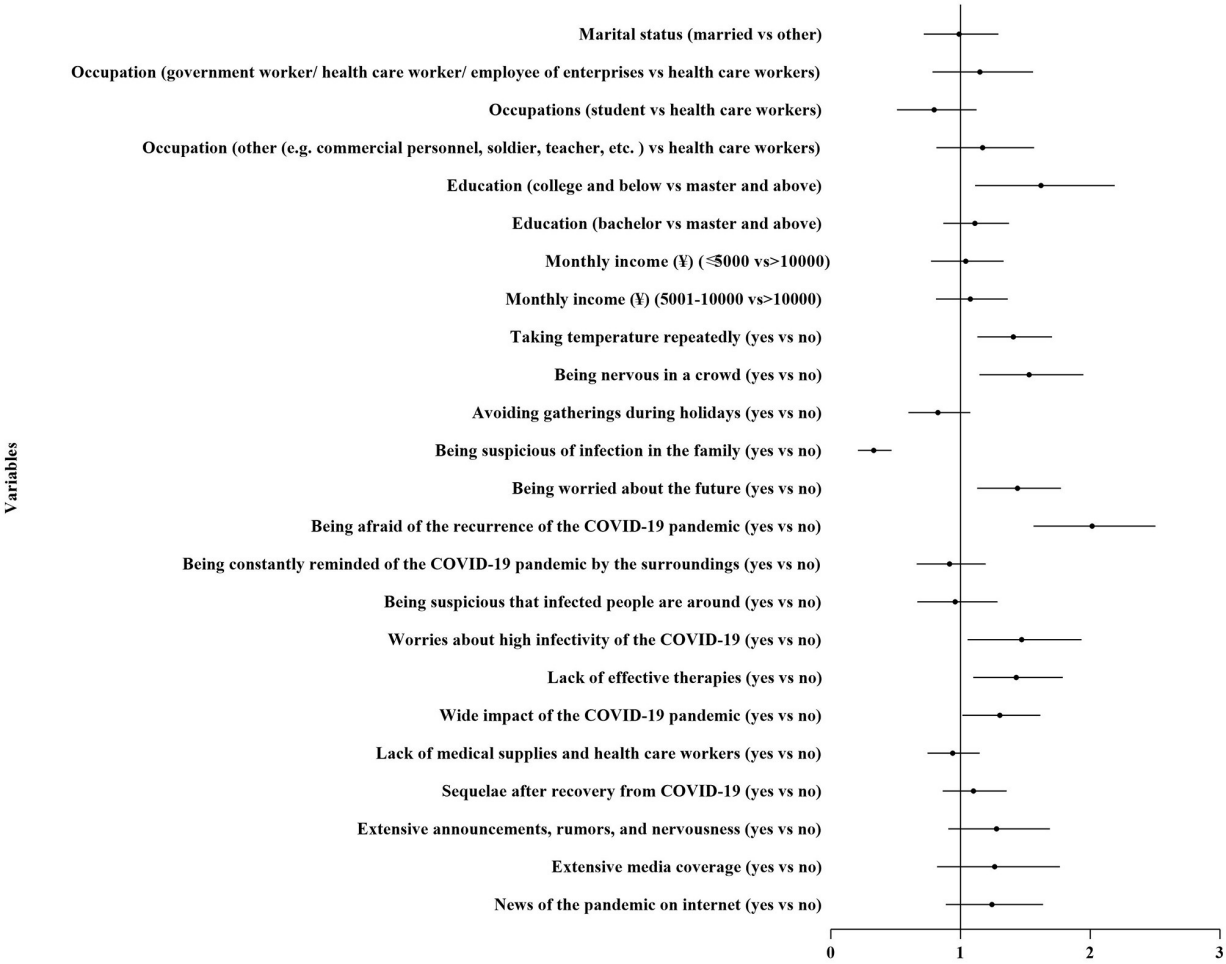
The forest plot of the risk factors and the associate factors of public panic.

The results from the multivariable logistic regression are presented in Table 2 and Figure 5. Psycho-behavioral responses, such as taking temperature repeatedly (odds ratio [OR] 1.388, 95% CI 1.130–1.706), being nervous in a crowd (OR 1.494, 95% CI 1.146–1.948), being suspicious of infection in the family (OR 1.416, 95% CI 1.128–1.775), and being worried about the future (OR 1.979, 95% CI 1.563–2.504) increased the chances of panic. Among those with worries about high infectivity of the COVID-19 (OR 1.429, 95% CI 1.055–1.934), lack of effective therapies (OR 1.402, 95% CI 1.099–1.789), and wide impact of the COVID-19 pandemic (OR 1.281, 95% CI 1.016–1.616) also increased the chances of panic. Inversely, avoiding gatherings during holidays (OR 0.313, 95% CI 0.209–0.469) decreased the chances of panic.

**Table 2:** The multivariable logistic regression analysis.

**Variables**	**OR**	**95%CI**
**Demographic characteristics**		
**Marital status (married vs. other)**	0.962	0.717–1.292
**Occupation**		
• Government worker/ employee of enterprises vs. health care workers	1.106	0.785–1.560
• Student vs. health care workers	0.757	0.510–1.124
• Other (e.g., commercial personnel, soldier, teacher, etc.) vs. health care workers	1.130	0.815–1.568
**Education**		
• College and below vs. master and above	1.561	1.113–2.190
• Bachelor vs. master and above	1.092	0.868–1.374
**Monthly income (¥^a^)**		
• ≤ 5,000 vs. >10,000	1.015	0.773–1.333
• 5,001–10,000 vs. >10,000	1.052	0.811–1.365
**Psycho-behavioral responses**		
**Taking temperature repeatedly** (yes vs. no).	1.388	1.130–1.706
**Being nervous in a crowd** (yes vs. no).	1.494	1.146–1.948
**Avoiding gatherings during holidays** (yes vs. no).	0.313	0.209–0.469
**Being suspicious of infection in the family** (yes vs. no).	1.416	1.129–1.775
**Being worried about the future** (yes vs. no).	1.979	1.563–2.504
**Being afraid of the recurrence of the COVID-19 pandemic** (yes vs. no).	0.890	0.663–1.194
**Being constantly reminded of the COVID-19 pandemic by the surroundings** (yes vs. no).	0.926	0.667–1.286
**Being suspicious that infected people are around** (yes vs. no).	0.802	0.598–1.076
**Risk perception of the COVID-19 pandemic**		
**Worries about high infectivity of the COVID-19** (yes vs. no).	1.429	1.055–1.934
**Lack of effective therapies** (yes vs. no).	1.402	1.099—.789
**Wide impact of the COVID-19 pandemic** (yes vs. no).	1.281	1.016–1.616
**Lack of medical supplies and health care workers** (yes vs. no).	0.925	0.746–1.148
**Sequelae after recovery from COVID-19** (yes vs. no).	1.082	0.863–1.356
**Extensive announcements, rumors, and nervousness** (yes vs. no).	1.238	0.907–1.690
**Extensive media coverage** (yes vs. no).	1.204	0.820–1.767
**News of the pandemic on the internet** (yes vs. no).	1.205	0.887–1.637

a *1 ¥ = US $0.15*.

## Discussion

### Principal Findings

Our study found that the prevalence of public panic was 23.39% during the earliest stage of the COVID-19 pandemic in China. The prevalence of panic was higher than the prevalence before the pandemic (5.6**–**13.2%) (44–47). The pandemic has seriously threatened people's daily life and greatly disrupted the normal social order, resulting in tremendous changes in population health, and inducing the prevalence of public panic. It has been found in recent studies that the pandemic can trigger the negative emotions, such as sadness, worry and fear, and increase public's vulnerability to mental disorders, such as panic, social anxiety, depression, and post-traumatic stress disorder (48–52). During this vital period, the examination of public panic and the associated factors are indispensable, but also scarce.

Results from our study indicate that panic is significantly associated with psycho-behavioral responses. The psycho-behavioral responses, such as taking temperature repeatedly, being nervous in a crowd, being worried about the future, being afraid of the recurrence of the COVID-19 pandemic, being suspicious of infection in the family, and being worried about the future, were the key risk factors of public panic. Whereas, avoiding gatherings during holidays could decrease the possibility of public panic. The public has paid more attention to personal protection because of the severe impacts of COVID-19 in China. It is common for the public to adopt social distancing measures, mask wearing, frequent hand washing, and canceling social activities during the pandemics to reduce disease spread (53, 54). While the public health measures were necessary to prevent the spread of COVID-19, the impact on public behaviors and risk perception damaged mental wellbeing (55, 56). Previous research also reported that social distancing behaviors, a common response to avoid infection, were associated with negative psychological responses (57, 58). The rapid changes during the COVID-19 pandemic raised problems in social order and development of finance, resulting in risks threatening people's health, stable income, and living quality, triggering further public panic and other mental disorders.

The results of our survey were in agreement with the findings in recent studies that during the public health emergencies, the disruption of daily routine, the uncertainty of epidemic, the threats to wellbeing, and the wide range of disease-related information were the main factors associated with psychological distress including fear, anxiety, depression, and panic (59–61). Strengthening the understanding of psychological responses and their impact on mental health, reducing public panic, and effectively managing irrational responses have become prominent issues that need to be resolved globally in order to reduce the detrimental impacts of the pandemic on people's psychological health. Therefore, psychological interventions and guidance on behaviors during public health emergencies should be developed and popularized among the public to help them regulate negative psychological changes and to cope with the pandemic with appropriately rational behaviors.

In addition, the public panic was associated with risk perception of the COVID-19 pandemic. In this study, worries about the high infectivity of the COVID-19, worried about lack of effective therapies, and wide impact of the COVID-19 pandemic have increased the public's risk perception of the COVID-19 threat. Cognitive responses are associated with behavior coping and perception of the pandemic (62, 63). Perceptions of the pandemic, such as the mode of transmission, symptoms and therapies of the disease, proper self-protective behaviors, the importance of early detection, and treatment of mental disorders, should be enhanced among the public through official channels (64–66). Besides, the worries about potential risks of tough treatment experience, huge medical cost, long time of recovery, delay of study plan, or unemployment raised from infection of COVID-19 might be part of the influencing factors of panic.

In addition to the variables as above, the outcomes of the chi-square test also indicated that the prevalence of panic was also higher among the participants who were concerned about sequelae after recovery from COVID-19; extensive announcements, rumors, and nervousness; extensive media coverage; and news of the pandemic on the internet. The methods of acquisition of health information were also significantly associated with poor mental wellbeing and public's knowledge and attitude about the COVID-19 pandemic (11, 67, 68). While the various sources of pandemic-related information provide convenience for the public to access information in a timely manner, the accuracy and authenticity of the health information were hard to guarantee, resulting in a wide diversity of psychological-behavioral responses, or even triggering public panic (69, 70).

The general public is vulnerable to excessively high-risk perception and uncoordinated behavioral responses during a public health emergency (71, 72). Negative psychological impacts raised by inappropriate risk perception of the COVID-19 pandemic can seriously affect the public's normal life and mental health (9, 10, 32). Although people should protect their health, they should also adjust their risk perceptions and enhance their coping mechanisms during epidemics (73, 74). A recent study found that <50% of the public had sufficient and appropriate risk perception of the COVID-19 pandemic to ensure their wellbeing (75). Educational programs could resolve the misconceptions about the COVID-19 pandemic (76). It is vital for the public to perceive the pandemic within a controllable range with reliable sources of health information and to cope with pandemic-related stressors in healthy ways with available instructions from psychological professionals during public health emergencies.

### Limitations

This study has several limitations. First, since the prevalence of panic is analyzed with a self-developed question, the generalizability of the outcomes is limited to other populations. Second, this survey was conducted during the earliest stage of the COVID-19 pandemic. Therefore, the collected data can only represent this special period, and the generalizability of the results to the other stages of the COVID-19 pandemic might be limited. Third, the results are limited by selection bias because the survey was conducted on the WeChat platform and the participants were only smartphone users. Finally, the results may be limited due to unmeasured confounders, such as gender-based violence and family violence.

## Conclusions

The public in mainland China has suffered from the high prevalence of public panic during the earliest stage of the COVID-19 pandemic. The public panic stemmed from a variety of factors, such as excessive self-protective behaviors, such as taking temperature repeatedly, being nervous in a crowd, being suspicious of infection around; and inappropriate risk perception of the COVID-19 pandemic, such as worries about high infectivity of the COVID-19, lack of effective therapies and wide impact of the COVID-19 pandemic. Timely prevention and screenings of panic should be enhanced for the public to keep their mental status stable and healthy. This could be accomplished by managing health information, guiding appropriate self-protective behaviors, strengthening health education in communities, and promoting treatments of mental disorders.

## Data Availability Statement

The raw data supporting the conclusions of this article will be made available by the authors, without undue reservation.

## Ethics Statement

The studies involving human participants were reviewed and approved by the Ethics Committee of China Medical University. The patients/participants provided their written informed consent to participate in this study.

## Author Contributions

WZ and CZ analyzed the data and drafted and revised the manuscript. KS revised the manuscript. CC, JF, SH, QP, and QC contributed to the acquisition and interpretation of data. XY was responsible for the conception and design and the revision of the manuscript. All authors contributed to the article and approved the submitted version.

## Funding

This study was conducted with the support from Research on Behaviors Rules and Emotions of the Public during the COVID-19 Epidemic, Public Behavior Research Project of Prevention and Control of COVID-19, and China Medical University (Grant Number: 121-1210120025).

## Conflict of Interest

The authors declare that the research was conducted in the absence of any commercial or financial relationships that could be construed as a potential conflict of interest.

## Publisher's Note

All claims expressed in this article are solely those of the authors and do not necessarily represent those of their affiliated organizations, or those of the publisher, the editors and the reviewers. Any product that may be evaluated in this article, or claim that may be made by its manufacturer, is not guaranteed or endorsed by the publisher.
